# Anxiety and depression among tunisian health professionals facing COVID-19

**DOI:** 10.1192/j.eurpsy.2021.817

**Published:** 2021-08-13

**Authors:** L. Aribi, R. Jbir, N. Messedi, O. Bouattour, Z. Elloumi, W. Bouattour, F. Charfeddine, J. Aloulou

**Affiliations:** 1 Psychiatry (b), Hedi Chaker University hospital, Sfax, Tunisia; 2 Psychiatry, Hedi chaker Sfax, sfax, Tunisia; 3 Psychiatry (b), Hedi Chaker University hospital, sfax, Tunisia; 4 Psychiatry, Hedi Chaker University hospital, sfax, Tunisia

**Keywords:** Depression, health professionals, Anxiety, COVID-19

## Abstract

**Introduction:**

In March 2020, the World Health Organization characterized the COVID-19 outbreak as a pandemic. This new health situation has created an anxiety-provoking climate, in particular among health professionals

**Objectives:**

To study the prevalence and predictors of anxiety and depression among health workers

**Methods:**

Our study was descriptive and analytical cross-sectional, carried out with healthcare on the period between May until June 2020. An anonymous online survey was sent to caregivers. The HADS questionnaire was used to screen for anxiety and depression

**Results:**

125 responses was collected The average age of the sample was 32 years. The participants were predominantly female (72.8%), married (48%), and had at least one child (39.2%). 21.6% of the participants worked in the resuscitation anesthesia service and urgent medical aid, 14.4% in the medical services at high risk of contamination, 1.6% in the COVID-19 unit Many changes in habits were reported by the participants: 28.7% had increased their consumption of coffee/tea, especially with anxious people (p = 0.001). This increase was also noted for tobacco (30.8%) and alcohol consumption (12.5%). According to the HADS scale, anxiety was retained in 44% and depression in 47.2%. Anxiety was significantly related to sex with (p = 0.039) and affects more women than men The consumers of coffee/tea developed more anxiety (p = 0.034) and depression (p = 0.026).

**Conclusions:**

This tragic health crisis had a major impact on the mental health of our heroes This is why we should better understand their vulnerability to psychological suffering to provide them with the necessary support

